# From Rough to Precise: Human-Inspired Phased Target Learning Framework for Redundant Musculoskeletal Systems

**DOI:** 10.3389/fnbot.2019.00061

**Published:** 2019-07-31

**Authors:** Junjie Zhou, Jiahao Chen, Hu Deng, Hong Qiao

**Affiliations:** ^1^State Key Laboratory of Management and Control for Complex Systems, Institute of Automation, Chinese Academy of Sciences, Beijing, China; ^2^School of Artificial Intelligence, University of Chinese Academy of Sciences, Beijing, China; ^3^Beijing Key Laboratory of Research and Application for Robotic Intelligence of “Hand–Eye–Brain” Interaction, Beijing, China; ^4^Research Center for Brain-Inspired Intelligence, Institute of Automation, Chinese Academy of Sciences, Beijing, China; ^5^CAS Center for Excellence in Brain Science and Intelligence Technology, Shanghai, China

**Keywords:** musculoskeletal system, human-inspired motion learning, noise in nervous system, reinforcement learning, phased target learning

## Abstract

Redundant muscles in human-like musculoskeletal robots provide additional dimensions to the solution space. Consequently, the computation of muscle excitations remains an open question. Conventional methods like dynamic optimization and reinforcement learning usually have high computational costs or unstable learning processes when applied to a complex musculoskeletal system. Inspired by human learning, we propose a phased target learning framework that provides different targets to learners at varying levels, to guide their training process and to avoid local optima. By introducing an extra layer of neurons reflecting a preference, we improve the Q-network method to generate continuous excitations. In addition, based on information transmission in the human nervous system, two kinds of biological noise sources are introduced into our framework to enhance exploration over the solution space. Tracking experiments based on a simplified musculoskeletal arm model indicate that under guidance of phased targets, the proposed framework prevents divergence of excitations, thus stabilizing training. Moreover, the enhanced exploration of solutions results in smaller motion errors. The phased target learning framework can be expanded for general-purpose reinforcement learning, and it provides a preliminary interpretation for modeling the mechanisms of human motion learning.

## 1. Introduction

Research on human-like musculoskeletal robots has become multidisciplinary in recent years, as it involves fields such as neuroscience and materials science for modeling and implementing musculoskeletal motor systems. In fact, this branch of robotics mainly comprises muscle models (actuators), skeletal systems (supporting structure), and methods for motion control and learning (control systems). Related work can roughly be divided into two types, namely, muscle dynamics modeling along with hardware design (Jäntsch et al., 2013; Kurumaya et al., 2016; Asano et al., 2017) and musculoskeletal robot control (Pennestrì et al., 2007; Jagodnik and van den Bogert, 2010; Tahara and Kino, 2010). Although most studies have been focused on the first type, the development of neuroscience has gradually increased the research on human-inspired control.

As a multibody mechanical system (Stoianovici and Hurmuzlu, 1996; Shi and McPhee, 2000) comprising muscles and joints, the human musculoskeletal system has several advantages. For instance, muscle redundancy maintains the reliable operation of the musculoskeletal system when some muscles are fatigued or even damaged. Under control of the central nervous system, the musculoskeletal system can accomplish accurate and fine manipulation (Rasmussen et al., 2001; Chen et al., 2018). To unveil the mechanisms that provide such advantages, Hill studied the contraction properties of muscles, establishing the Hill model (Hill, 1938). From this fundamental work, a series of muscle dynamic models have been proposed (Huxley and Niedergerke, 1954; Eisenberg et al., 1980; Zahalak and Ma, 1990), but all of them present specific limitations. For instance, the simple second-order model (Cook and Stark, 1968; Agarwal et al., 1970) lacks independent nodal locations for external input signals, which indirectly affect the output. The Huxley contraction model (Huxley, 1957) is highly complex and no general-purpose method has been developed to obtain its parameters (Winters and Stark, 1987). The Hill model presents difficulties in measuring the fiber length during motion (Arnold and Delp, 2011).

Research has also been devoted to design hardware for emulating muscle characteristics. The Anthrob muscle unit (Jäntsch et al., 2013) and the sensor–driver integrated muscle module (Asano et al., 2015) try to resemble muscular structures. However, the weight and size of motors make hardware models notably diverge from biological muscles. Furthermore, resembling tiny human muscles through hardware design is difficult, thus undermining their applicability. In materials science, the synthesis of ideal materials for artificial muscles is being pursued to achieve the characteristics of biological muscles regarding size, weight, stiffness, and dynamic behavior. New materials for artificial muscles usually share some problems, including unsafe voltages and low strain. Accessory equipment can partly adjust the characteristics of materials. For instance, liquid-vapor transition has been used on a soft composite material (Miriyev et al., 2017) for implementation as an actuator in a variety of robotic applications. In addition, a coiled polymer muscle (Haines et al., 2014) controlled by varying water temperature prevents dependence on electricity. Hence, advanced design methods and materials seem promising to develop artificial muscles that closely reflect the dynamics of their biological counterparts.

Based on the abovementioned models, control systems developed for musculoskeletal robotics also face challenges. Redundant muscles and extremely complex tendon forces impose several barriers for direct solutions of muscle excitation. Widely used methods, such as inverse dynamics with static optimization (Crowninshield and Brand, 1981), computed muscle control (Thelen et al., 2003), proportional-derivative control (Jagodnik and van den Bogert, 2010), and PI-type iterative learning control (Tahara and Kino, 2010), are used to regulate musculoskeletal systems. Although some conventional methods, such as computed muscle control, theoretically compute muscle excitation signals, they also demand intensive computations for sophisticated processes (Chen et al., 2018). In addition, these control strategies are hardly supported by biological evidence showing that they resemble the approach of human motion learning.

In recent years, reinforcement learning has become a popular control method in robotics as it provides a natural-like approach to learn from the environment. In fact, as a method that fosters interaction with uncertain environments, reinforcement learning allows a learner to observe the environment and then execute appropriate actions. The environment provides rewards for each action, and the learner aims to maximize its rewards during decision-making. This learning process is similar to that of humans and animals (Sutton and Barto, 2018). Studies in neuroscience (Schultz et al., 1997; Law and Gold, 2009) verify this principle, and hence it is reasonable to consider human-like learning from the viewpoint of reinforcement learning (Tesauro, 1995; Diuk et al., 2008; Riedmiller et al., 2009). Deep neural networks are adopted to implement reinforcement learning. Specifically, the deep Q-network (Mnih et al., 2015) uses a deep convolutional neural network to estimate the action-value function, making deep reinforcement learning a powerful weapon for a myriad of applications (Van Hasselt et al., 2016; Wang et al., 2016; Hou et al., 2017). However, when applied to the musculoskeletal system, the performances of deep neural networks can be unstable. Given muscle redundancy in the musculoskeletal system, the additional dimensions expand the solution space, hindering optimization through reinforcement learning.

In this study, we focused on the unstable training of musculoskeletal systems and the expanded solution space of excitations to provide three contributions. (1) The learning goal of humans, changes stepwise as learning proceeds over advancing levels. For example, running requires higher physical coordination than walking, and one cannot run before learning to walk. Thereby, the learner target evolves from walking to running during this process. Based on this principle, we propose the phased target learning (PTL) framework that reduces the computational cost for exploration in a high-dimensional solution space. In addition, phased targets guide the convergence of excitations to the expected value during training. (2) As sensory information may be encoded by opposite tuning neurons (Romo and de Lafuente, 2013), we improve an MLP-based Q-network by introducing an extra layer of neurons reflecting preference and using various relative action probabilities from value functions for obtaining continuous outputs to control a musculoskeletal arm model. (3) As noise exists in the nervous system (A Aldo et al., 2008) and based on information transmission in the human nervous system (Dhawale et al., 2017), we introduce two noise sources at the sensor and execution levels into the proposed PTL framework. These noise sources increase the exploration capacity in the solution space during training and strengthen the control robustness.

In this paper, in section 2, we introduce the muscle dynamics, the structure of the arm model, and detail the musculoskeletal system considered in this study. Moreover, optimization of the proposed PTL framework is outlined. Then, the PTL framework with the biological noise sources is introduced in section 3. Experimental results and conclusions are presented in sections 4 and 5, respectively.

## 2. Musculotendon Model and Musculoskeletal Arm Model

Modeling muscles is difficult because most parameters cannot be measured precisely in real time (Arnold and Delp, 2011). According to the Hill model (Hill, 1938), which defines that a muscle is made up of separate elements, such as contractile elements (CE), passive elements (PE), and series elastic elements (SEE) (Zajac, 1989; Thelen et al., 2003), we design a control framework for musculoskeletal systems.

### 2.1. Musculotendon Model

To determine the way a human can control complex muscle systems, a muscle dynamic model is necessary. Let *u* ∈ [0, 1] denote an idealized muscle excitation signal. According to a nonlinear first-order differential Equation (1), muscle activation signal *a* can be computed (Thelen, 2003):

(1) $$\begin{array}{r} \frac{da}{dt}=\frac{u\;-\;â}{\tau \left(u,a\right)}\;, \end{array}$$

where τ varies according to idealized muscle excitation signal *u* and activation signal *a* (Winters, 1995), â is the activation signal after normalization, and *a* is transmitted to the muscle contraction dynamic model as a final control signal.

**Figure 1:**
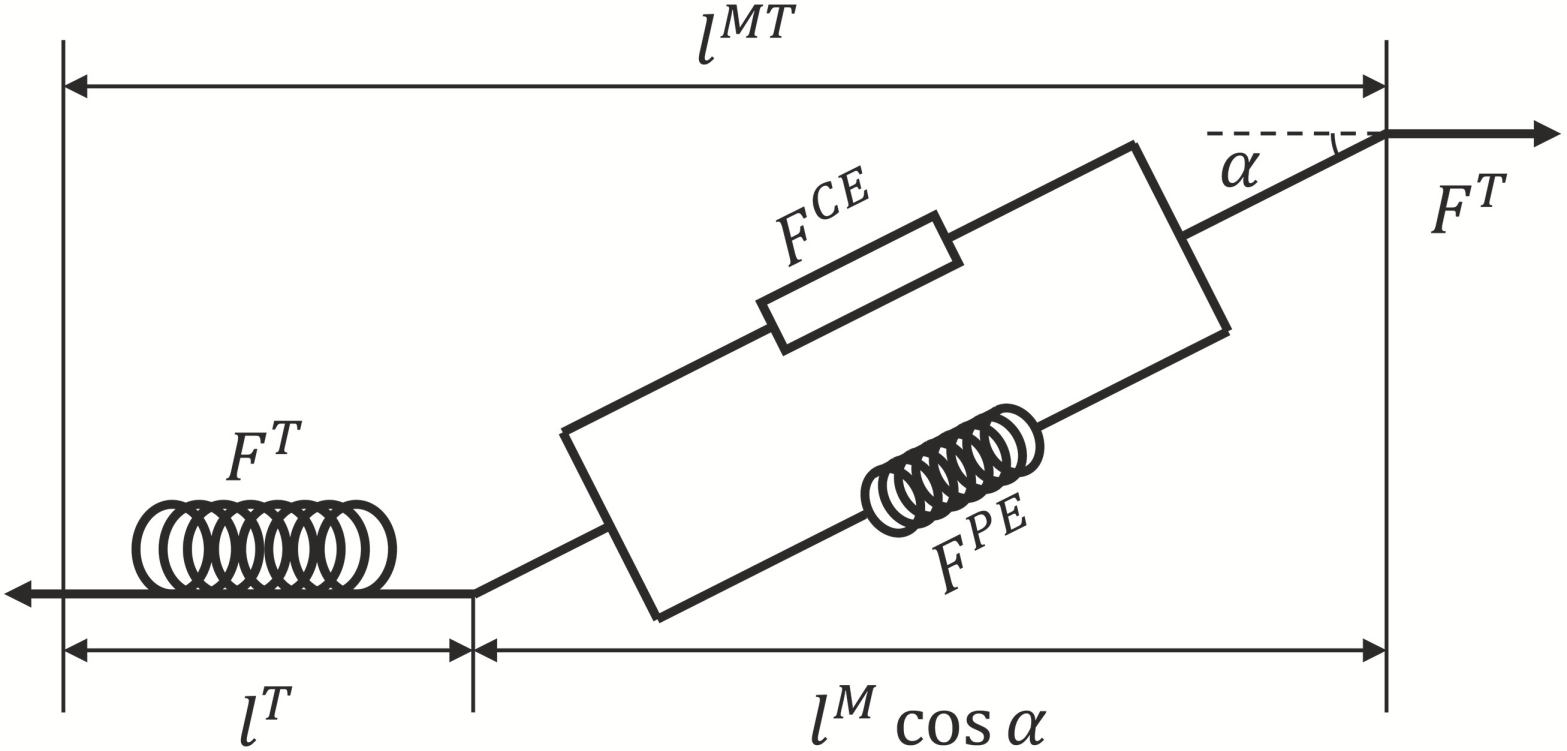
Structure of a Hill-type muscle model. *F*^*CE*^ and *F*^*PE*^ are the active and passive forces, respectively, and *F*^*T*^ is the tendon force (Hill, 1938; Thelen, 2003).

Before introducing the muscle contraction dynamics, the structure of a Hill-type muscle model is shown in Figure 1, where *l*^*T*^ and *l*^*M*^ are the lengths of the tendon and muscle fiber, respectively, and α is the muscle pennation angle (Garner and Pandy, 2003). When the activation signal *a* is transmitted to the muscle, the corresponding muscle force is generated by contraction. Then, the muscle force pulls the skeletons to generate motion or to maintain the balance of forces.

Suppose that signal *u* is known. To calculate tendon force *F*^*T*^, some assumptions are required. First, *F*^*T*^, *F*^*CE*^, *F*^*PE*^ > 0 because muscles move the skeleton by tension instead of thrusting. Second, the change of muscle width can be ignored during muscle contraction (Matthew et al., 2013). Third, muscle mass can be ignored. Using these assumptions, the dynamics of muscles can be described. Specifically, a pennation angle α can be obtained from

(2) $$\begin{array}{r} l_{s}^{M}\text{ sin}\left(\alpha_{0}\right)=l^{M}\left(t\right)\text{ sin}\left(\alpha \left(t\right)\right)\;, \end{array}$$

where $l_{s}^{M}$ and α_0_ are the slack length of a muscle fiber and initial pennation angle, respectively, which also define the initial muscle width, *l*^*M*^(*t*) and α(*t*) are the length of the muscle fiber and pennation angle at time *t*, respectively. From α(*t*), tendon force *F*^*T*^ can be computed by a piecewise nonlinear equation (Proske and Morgan, 1987; Thelen, 2003). In addition, the contraction velocity of a muscle fiber is necessary for the model. To determine this velocity, active force *F*^*CE*^ produced by the contractile element should be obtained first. According to the geometric relationship between tendon and muscle fiber (Figure 1), *F*^*CE*^ can be calculated indirectly as follows:

(3) $$\begin{array}{r} F^{CE}=\frac{F^{T}}{\cos\left(\alpha \right)}-F^{PE}\;, \end{array}$$

where *F*^*PE*^ is the passive force of the muscle fiber. During simulations, the muscle length sometimes causes numerical problems that result in *F*^*CE*^ < 0, which clearly violate the first assumption about muscles. Therefore, a constraint should be added to avoid exceptional cases:

(4) $$\begin{array}{r} F^{CE}=\max\left\{F^{CE},0\right\}\;. \end{array}$$

Then, contraction velocity *v*^*M*^ can be computed by another piecewise non-linear equation (Matthew et al., 2013):

(5) $$\begin{array}{l} v^{M}=f_{v}^{-1}\left(\frac{F^{CE}}{af_{l}(l^{M})}\right)\;, \end{array}$$

where *f*_*v*_ is the force–velocity function, $f_{v}^{-1}$ is its inverse function, and *f*_*l*_ is a Gaussian function with variable *l*^*M*^ (Winters, 1990). As a key variable in the muscle dynamics model, *v*^*M*^(*t*) affects *l*^*M*^(*t* + 1) at every timestep. Variable *l*^*M*^ is the fiber length and *l*^*MT*^ is the muscle length, which comprises fiber and tendon. Length *l*^*M*^ can be calculated directly using *v*^*M*^ and *F*^*T*^, whereas *l*^*MT*^ can be measured. Consequently, if signal *u*(*t*) is known, the contraction states of the muscle and tendon force *F*^*T*^(*t*) can be computed.

### 2.2. Musculoskeletal Arm Model

In the remainder of this section, we first establish a simplified arm model to connect muscles and bones. Then, we analyze the kinematic relationship between the arm model and muscle model. Finally, a control framework is outlined using this relationship.

**Figure 2:**
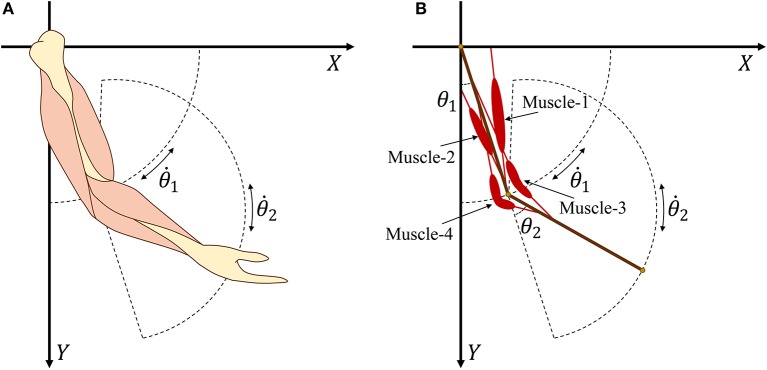
**(A)** Human arm model with four main muscles. **(B)** Simplified arm model with four muscles.

According to the Newton–Euler equation (Zixing, 2000; Hahn, 2013), we establish a two degree-of-freedom model (Figure 2) that consists of two segments and four muscles. Then, expected torque τ_*n*_ at the joints can be calculated as

(6) $$\begin{array}{l} \tau_{n}=\frac{\partial W}{\partial \theta }=M(\theta )\ddot{\theta }+C(\theta ,\dot{\theta })\dot{\theta }+G(\theta )\;, \end{array}$$

where *W* is the work from external forces, $\overset{∙}{\theta }$ is the vector of rotational velocity, $\dot{\theta }$ is the vector of rotational acceleration, *M*(θ) ∈ ℝ^*n*×*n*^ and $C(\theta ,\dot{\theta })\dot{\theta }\in {\mathbb{R}}^{n}$ is the inertia matrix and the centripetal and Coriolis force, respectively, and *G*(θ) ∈ ℝ^*n*^ is the gravitational force vector of our model. During forward calculation, Equation (6) provides a way to compute expected torques for known motion states. During inverse calculation, it can be used to compute actual angular acceleration.

### 2.3. Musculotendon Model Into Arm Model

In this section, we obtain the relationship between torques and motion states and define the adopted learning approach.

**Figure 3:**
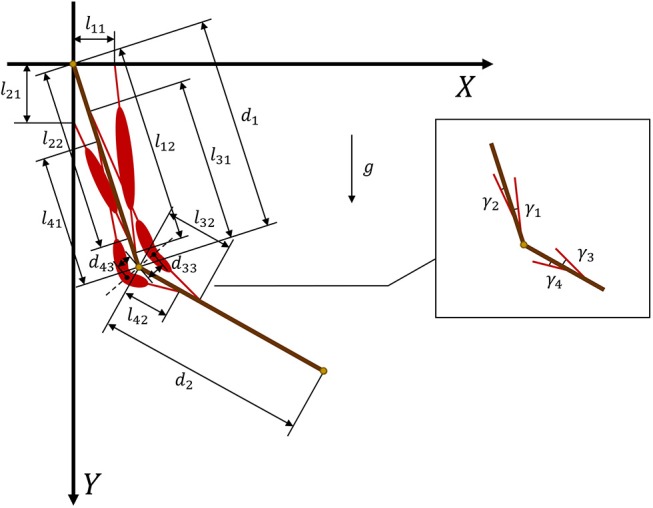
Geometry and parameters of musculoskeletal arm model. Muscles 1 and 2 are defined by straight lines, whereas muscles 3 and 4 are defined by polylines.

Unlike conventional robots that use a single joint motor to generate torque, each joint in a musculoskeletal system is usually affected by more than one muscle. Let τ_*i*_ be the muscle torque generated by muscle *i*:

(7) $$\begin{array}{r} \tau_{i}=F_{i}^{T}l_{i2}\text{ sin }\gamma_{i},\;i=1,2,\ldots ,n\;, \end{array}$$

where $F_{i}^{T}$ is the tendon force of muscle *i* and γ_*i*_ is the angle between the muscle and related bone. Figure 3 provides geometric details of the muscles and bones. We set *m*_1_ = 2 and *d*_1_ = 0.3 as the mass and length of the upper arm, respectively, whereas *m*_2_ = 1.8 and *d*_2_ = 0.3 are the mass and length of the forearm, respectively. For the given geometry of the musculoskeletal model, the muscle torque can be written as

(8) $$\{\begin{array}{l} \tau_{n1}^{\prime }=\tau_{1}-\tau_{2}=F_{1}^{T}l_{12}\sin\gamma_{1}-F_{2}^{T}l_{22}\sin\gamma_{2}\\ \tau_{n2}^{\prime }=\tau_{3}-\tau_{4}=F_{3}^{T}l_{32}\sin\gamma_{3}-F_{4}^{T}l_{42}\sin\gamma_{4}. \end{array}$$

In addition, the geometric parameters can be used to compute sin γ_*i*_:

(9) $$\begin{array}{l} \{\begin{array}{ll} \sin\gamma_{1}= & \frac{l_{11}\cos\theta_{1}}{\sqrt{l_{11}^{2}+l_{12}^{2}-2l_{11}l_{12}\sin\theta_{1}}}\\ \sin\gamma_{2}= & \frac{l_{21}\sin\theta_{1}}{\sqrt{l_{21}^{2}+l_{22}^{2}-2l_{21}l_{22}\cos\theta_{1}}}\\ \sin\gamma_{3}= & \frac{d_{33}\cos\frac{\theta_{2}}{2}}{\sqrt{l_{32}^{2}+d_{33}^{2}-2l_{32}d_{33}\sin\frac{\theta_{2}}{2}}}\\ \sin\gamma_{4}= & \frac{d_{43}\cos\frac{\theta_{2}}{2}}{\sqrt{l_{42}^{2}+d_{43}^{2}+2l_{42}d_{43}\sin\frac{\theta_{2}}{2}}} \end{array}\;. \end{array}$$

In muscles 3 and 4 (Figure 2), we introduce two turning points at the angular bisector of the elbow to design polyline muscles, where *d*_33_ and *d*_43_ are the distances from the elbow to the turning points of muscles 3 and 4, respectively. From Equation (9), it is clear that sinγ_*i*_ is a nonlinear function of θ_*i*_. By substituting Equation (9) into Equation (8), we obtain muscle torque functions $\tau_{n1}^{\prime }\left(F_{1}^{T},F_{2}^{T},\theta_{1}\right)$ and $\tau_{n2}^{\prime }\left(F_{3}^{T},F_{4}^{T},\theta_{2}\right)$.

For the muscle description in our arm model, it is difficult to determine its inverse function, because *F*^*T*^ and Equation (5) are piecewise functions with complicated expressions. Therefore, we usually cannot calculate *u*_*i*_ by directly using muscle force, but instead we adopt an indirect method.

We assume that expected states $\theta_{i},{\dot{\theta }}_{i}$ and ${\ddot{\theta }}_{i}$ are given. Expected torque τ_*n*_ can be calculated by Equation (6) as a learning target. On the other hand, actual tendon force *F*^*T*^ is known when corresponding excitation signals *u* are generated, and hence actual torque ${\tau^{\prime }}_{n}$ is calculable. To obtain actual angular accelerations ${\ddot{\theta }}_{i}$, Equation (6) can be computed reversely. In general, $\ddot{\theta }$ can be rewritten as $\ddot{\theta }\left(\tau_{n},\theta ,\dot{\theta }\right)$. Considering $\dot{\theta }=\frac{d\theta }{dt}$ and $\ddot{\theta }=\frac{d\dot{\theta }}{dt}$, joint angle θ at time (*t* + 1) can be obtained as

(10) $$\begin{array}{r} \theta_{t+1}\left(\tau_{n},\theta_{t}\left({\ddot{\theta }}_{t-1}\right),{\dot{\theta }}_{t}\left({\ddot{\theta }}_{t-1}\right),{\ddot{\theta }}_{t}\right)\;. \end{array}$$

If tendon force vector *F*^*T*^ satisfies

(11) $$\begin{array}{r} \tau_{n}\left(\theta ,\dot{\theta },\ddot{\theta }\right)=\tau_{n}^{\prime }\left(F^{T},\theta \right)\;, \end{array}$$

we can rewrite Equation (11) as

(12) $$\begin{array}{r} \theta_{t+1}\left(\tau_{n}^{\prime }\left(F_{t}^{T},\theta_{t}\right),\theta_{t}\left({\ddot{\theta }}_{t-1}\right),{\dot{\theta }}_{t}\left({\ddot{\theta }}_{t-1}\right),{\ddot{\theta }}_{t}\right)\;. \end{array}$$

The purpose of our framework is to find appropriate excitation signals *u* to generate tendon forces that satisfy Equation (11). As a result, the expected motions will be generated during exploration. Based on Equation (12), we establish a training framework for the musculoskeletal arm model. When excitation signal *u* is given, corresponding activation signal *a* and tendon force *F*^*T*^ can be calculated by muscle dynamics. Then, new motion states can be solved using the arm model. If excitation signal *u* is unknown, we should explore candidate solutions to generate *F*^*T*^ satisfying (Equation 11).

## 3. Human-Inspired Phased Target Learning Framework

**Figure 4:**
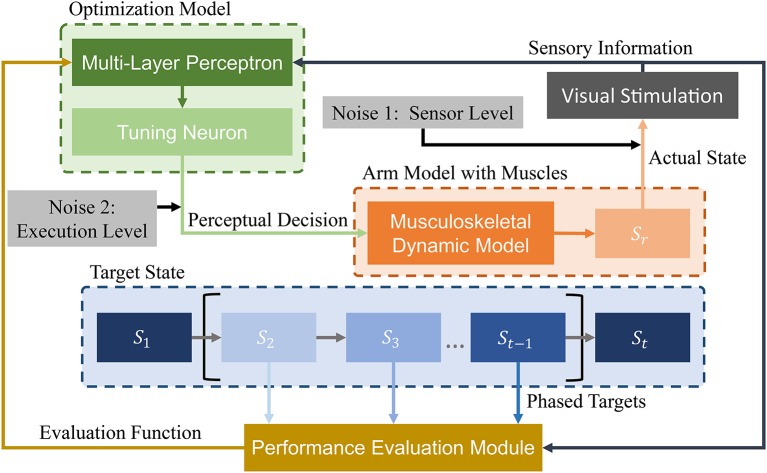
Schematic of proposed PTL framework for motion control of musculoskeletal robots. A vision sensor collects motion information. Then, visual stimuli are transmitted to the optimization model and performance evaluation module. During optimization, state information is processed by a multi-layer perceptron. Then, perceptual decisions (excitation signals) are transmitted to the arm model as optimization results. During performance evaluation, different phased targets are designed to guide arm motion states. Finally, the evaluation results are transmitted to the optimization model for improved decision-making. In addition, two biological noise sources are considered during learning for improved exploration ability.

We design a learning framework to solve signal *u*_*i*_. Conventional learning frameworks use expected states as the learning target. However, these targets can cause unforeseen problems during the solving process, and solutions can fall into local optima. In contrast, the proposed PTL framework can avoid local optima by guiding the learning process. Specifically, different learning targets are designed according to the learner's level, additionally providing high efficiency during training. We consider the musculoskeletal system, optimization model, and expected target state as the most essential aspects in our framework (Figure 4) and detail the last two parts in the sequel.

### 3.1. Phased Target Learning

#### 3.1.1. Simplified Target Setup

Consider a beginner who starts to learn dancing or practicing a sport. It is difficult for him to acquire all the professional postures and skills at once. Instead of trying to enhance memory or learning skills, the simplest solution is reducing the quality requirements and perform intensive practice through gradual improvement. This way, the beginner will easily improve by establishing simple learning targets that are gradually set at different levels as learning proceeds. In this study, we calculated precise motion states to be expected targets. Then, we designed different simplified states as easier targets for learning. Formally, let s∈S be the expected states of the arm model, and $s_{T}\in S_{T}$ be the simplified states. *s*_*T*_ can be calculated by simplifying *s*:

(13) $$\begin{array}{r} s_{T}\left(t\right)=s\left(ceil\left(\frac{t}{d}\right)\cdot d\right)\cdot \delta \left(0\right)\;,t=1,2,\ldots ,T\;, \end{array}$$

where δ(*t*) is an impulse function, and *d* ∈ ℕ_+_ satisfying $\frac{d}{T}\in \left[\frac{1}{T},1\right]$ is a forgetting factor. When *d* = *T*, *s*_*T*_ only reflects the endpoint state of expected state *s*, and when *d* = 1, *s*_*T*_ = *s*, indicating that *s*_*T*_ reflects all the states of S.

Obviously, simplification induces errors with respect to expected states. Suppose that θ∈S is the expected joint angle of the arm model, and $\theta_{T}\in S_{T}$ is the simplified joint angle. Then, we define the average allowed error between *s* and *s*_*T*_ as

(14) $$\begin{array}{r} {\bar{e}}_{T}=\frac{1}{T}\overset{T}{\underset{t=1}{\sum }}|\theta \left(t\right)-\theta_{T}\left(t\right)|\;. \end{array}$$

**Figure 5:**
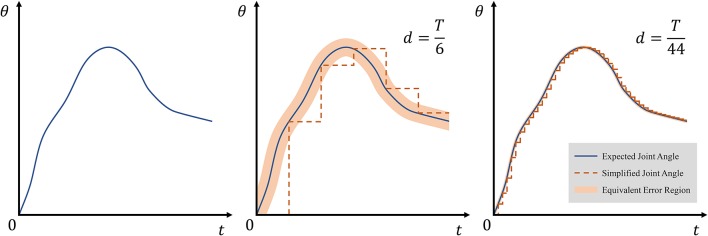
Effect of forgetting factor *d* on equivalent error region. The thin blue line represents expected angles θ, and the dotted orange line represents simplified state θ_*T*_. The equivalent error region is depicted with light orange and obtained as $\overset{T}{\underset{t=1}{\sum }}|\theta \left(t\right)-\theta_{T}\left(t\right)|$.

According to Equations (13) and (14), average allowed error ${\bar{e}}_{T}$ depends only on the forgetting factor *d*. Geometrically, ${\bar{e}}_{T}$ can be considered as the width of the equivalent error region. Figure 5 shows the width and effect of *d* on simplified joint angle curve θ_*T*_.

PTL provides different simplified targets for learning at varying training phases. When the motion accuracy achieves the average allowed error range, ${\bar{e}}_{T}$, a new and smaller average allowed error range is given to guide training. Then, we define actual average error ${\bar{e}}_{R}$ of motion as

(15) $$\begin{array}{r} {\bar{e}}_{R}=\frac{1}{T}\overset{T}{\underset{t=1}{\sum }}|\theta_{T}\left(t\right)-\theta_{R}\left(t\right)|\;. \end{array}$$

Unlike Equation (14), Equation (15) uses the actual joint angle, θ_*R*_. In addition, ${\bar{e}}_{T}$ is updated after each training iteration. A new average allowed error is computed only when

(16) $$\begin{array}{r} {\bar{e}}_{T}-{\bar{e}}_{R}>0\;. \end{array}$$

(17) $$d=\{\begin{array}{ll} D(d), & {\bar{e}}_{T}-{\bar{e}}_{R}>0,D(d)\;⩾1;\\ 1, & {\bar{e}}_{T}-{\bar{e}}_{R}>0,D(d)<1;\\ d, & {\bar{e}}_{T}-{\bar{e}}_{R}\;⩽0; \end{array}$$

Equation (17) is the update rule of forgetting factor *d*, where *D*(*d*) is a function that satisfies *D*(*d*) < *d*. It is convenient to maintain the value of |*d* − *D*(*d*)| small, because a large difference between adjacent simplified states vanishes the gradual learning effect.

#### 3.1.2. Performance Evaluation Function

Conventional temporal-difference learning methods are highly suitable for model-free learning. Considering Equation (11), the inverse function of $\tau_{n}^{\prime }$ should be determined and can be set as a model-free problem. In this study, we aimed to improve the Q-network to estimate the continuous excitation signal *u* for musculoskeletal systems. Then, we combined it with PTL to calculate appropriate control signals.

Let *T* be the number of finite timesteps and *u*_*i*_ be the excitation signal for muscle *i*. Each signal *u*_*i*_(*t*) at time *t* has two possible actions; either increase [*a*_*i*,1_(*t*)] or decrease [*a*_*i*,2_(*t*)]. The adjustment of *u*_*i*_ affects the muscle and musculoskeletal model at time (*t* + 1).

However, the two actions only determine the increment sign, and additional parameters are required to calculate the step sizes. Furthermore, the difference between adjacent states can hinder perceptron learning from input states during training. Moreover, incorrect adjustments can lead to signal oscillation in the redundant musculoskeletal model.

In human cortical circuits, sensory information is encoded by neurons via opposite tuning (Romo and de Lafuente, 2013). Based on this mechanism, we redefine action-value function *Q*_*u*_*i*_, *j*_ as a probability of signal *u*_*i*_ executing action *a*_*i,j*_. Equation (18) defines *u*_*i*_ as

(18) $$\begin{array}{l} u_{i}=\frac{1}{\sum_{J=1}^{2}Q_{u_{i},j}}(Q_{u_{i},1}u_{\max}+Q_{u_{i},2}u_{\min}),\;i=1,2,...,n \end{array}$$

and action-value function *Q*_*u*_*i*_, *j*_ is redefined as

(19) $$\begin{array}{l} Q_{u_{i},j}(s_{t},a_{i,j,t})=𝔼\left[E_{u_{i}}(s_{t+1},a_{i,j,t+1})+\gamma Q_{u_{i},j}(s_{t+1},a_{i,j,t+1})\right],\\ \;i=1,2,...,n;\;j=1,2, \end{array}$$

where *E*_*u*_*i*__ is an evaluation function related to arm motion. According to Equations (18) and (19), a specific action value of a function is not enough to obtain the excitation signal in our method. Instead, relative values of different functions determine an excitation signal, and thus *Q*_*u*_*i*_, 1_ and *Q*_*u*_*i*_,2_ should be maintained balanced. In addition, note that *E*_*u*_*i*__ is used in Equation (19) instead of conventional reward function *R*_*u*_*i*__. This is because the *R*_*u*_*i*__ is a decreasing function of the action error, and during training, reducing action errors increases *R*_*u*_*i*__ and *Q*_*u*_*i*_, *j*_. In this case, the balance of action-value functions is affected by increasing *Q*_*u*_*i*_, *j*_. Therefore, we employ evaluation function *E*_*u*_*i*__, which is an increasing function of the action error. Reducing errors therefore imply smaller *E*_*u*_*i*__ and a weaker effect than *R*_*u*_*i*__ on the balance of action-value functions. Furthermore, (*E*_*u*_*i*__)_min_ > 0 promotes stability, as detailed in section 3.1.3.

We obtain the performance evaluation function as follows:

(20) $$\begin{array}{r} E_{u_{i}}\left(e_{R}\right)=p\cdot \exp\left[m\cdot g^{2}\left(e_{R}\right)\right]+k \end{array}$$

(21) $$\begin{array}{r} g\left(e_{R}\right)=\min\left[|e_{R}|,e_{0}\right]\;, \end{array}$$

where *p, m, k* > 0 are parameters of *E*_*u*_*i*__ and function *g*(*e*_*R*_) prevents exploding gradients under large errors.

#### 3.1.3. Learning by Gradient Descent

We define the loss function by summing the squared errors between expected action value $Q_{u_{i},j}^{\prime }$ and actual action value $E_{u_{i},j}+\gamma Q_{u_{i},j}^{\prime }$:

(22) $$\begin{array}{l} L(\theta )=\frac{1}{2}𝔼\left[\sum_{i=1}^{n}\sum_{j=1}^{2}{\left(E_{u_{i},j}+\gamma {Q^{\prime }}_{u_{i},j}(s^{\prime },a^{\prime };\theta^{\prime })-Q_{u_{i},j}(s,a;\theta )\right)}^{2}\right]\;, \end{array}$$

where γ is a factor to discount the future action value. The gradient of the loss function is given by

(23) $$\begin{array}{l} ▽L(\theta )=𝔼[\sum_{i=1}^{n}\sum_{j=1}^{2}\gamma \left(E_{u_{i},j}+\gamma {Q^{\prime }}_{u_{i},j}(s^{\prime },a^{\prime };\theta^{\prime })-Q_{u_{i},j}(s,a;\theta )\right)\\ \;▽{Q^{\prime }}_{u_{i},j}(s^{\prime },a^{\prime };\theta^{\prime })]\;. \end{array}$$

During backpropagation, the outputs of multi- layer perceptron in our model can be easily obtained. We suppose that $Q_{u_{i},j}^{\prime }$ represents the result of the output layer and can be expressed as

(24) $$\begin{array}{l} {Q^{\prime }}_{u_{i},j}=f\left(\sum_{h=1}^{n_{h}}\omega_{hk}y_{h}\right)\;, \end{array}$$

where *f*(*x*) is the sigmoid activation function, ω_*hk*_ is the weight of the edge from the *h*-th node in the hidden layer to the *k*-th node in the output layer. Consider ω_*hk*_ as an example, the weight increment is given by

(25) $$\begin{array}{rc} △\omega_{hk} & =-\eta \frac{\partial L}{\partial \omega_{hk}} \end{array}$$

(26) $$\begin{array}{r} =-\eta \overset{n}{\underset{i=1}{\sum }}\overset{2}{\underset{j=1}{\sum }}\gamma \left(E_{u_{i},j}+\gamma Q_{u_{i},j}^{\prime }-Q_{u_{i},j}\right)\frac{\partial Q_{u_{i},j}^{\prime }}{\partial \omega_{hk}} \end{array}$$

(27) $$=-\eta \sum_{i=1}^{n}\sum_{j=1}^{2}\gamma (E_{u_{i},j}+\gamma Q_{u_{i},j}^{\prime }-Q_{u_{i},j})f^{\prime }\left(\sum_{h=1}^{n_{h}}\omega_{hk}y_{h}\right)\sum_{h=1}^{n_{h}}y_{h}\;,$$

where *y*_*h*_ is the output of the *h*-th node in the hidden layer. When the excitations become stable, the expected increment is Δω_*hk*_ → 0 such that Δ*Q*_*u*_*i*_, *j*_ → 0, and hence $E_{u_{i},j}+\gamma Q_{u_{i},j}^{\prime }=Q_{u_{i},j}$ at this time. Factor γ is known as a decimal, and we can infer $\gamma Q_{u_{i},j}^{\prime }<Q_{u_{i},j}$, which explains why the performance evaluation function should satisfy (*E*_*u*_*i*_,*j*_)_min_ > 0.

### 3.2. Noise in Nervous System

Noise is ubiquitous in real-world systems, especially during information transmission. As motion learning consists of information transmission, noise is present. Recent research roughly identified noise sources in the nervous system at the sensor and action levels (A Aldo et al., 2008). We considered these noise sources in the proposed PTL framework.

#### 3.2.1. Noise at Sensor Level

During the collection of visual information, photoreceptors receive photons reflected by objects under the influence of Poisson noise, which reduces the accuracy of optical information (Bialek, 1987). Although sensory noise is inevitable (Bialek and Setayeshgar, 2005), it also mitigates sensitivity of the redundant musculoskeletal system.

When motion tracking is performed on the redundant musculoskeletal arm model, the Q-network method can exhibit unstable training, because joint angles are affected by the action of many muscles, likely falling into local optima. Then, any small fluctuation of excitation signals can be amplified and cause divergent signals. However, when target motion is considered as a region, fluctuations are tolerated. We use Poisson noise to conform tolerance regions and prevent rapid fluctuations:

(28) $$\begin{array}{r} s_{RN}=s_{R}+N_{1}\;, \end{array}$$

(29) $$\begin{array}{r} N_{1}\sim Pois\left(\lambda \right)\;, \end{array}$$

where *s*_*R*_ is the actual arm state, *s*_*RN*_ is the observed arm state observed by the vision sensor, and *N*_1_ is Poisson noise in the visual information. In our algorithm, let *s*_*R*_ = *s*_*RN*_ represents the inputs of the improved Q-network.

#### 3.2.2. Noise at Execution Level

Noise at the sensor level is also called planning noise, as it affects decision-making. In addition, execution noise exists and is superimposed on the original decision signals. In fact, execution noise describes an uncontrollable noise whose standard deviation is linearly related to the mean muscle force (Hamilton et al., 2004; Dhawale et al., 2017) and can be expressed as

(30) $$\begin{array}{r} u_{Ni}=\min\left[\max\left[u_{i}+N_{2},0\right],1\right]\;, \end{array}$$

where $N_{2}\sim N\left(0,{\left(vF^{T}\right)}^{2}\right)$ simulates noise in the motor system periphery, *u*_*i*_ and *u*_*Ni*_ are undisturbed and noisy signals from perceptron, respectively, and *v* is a scale coefficient of tendon force *F*^*T*^. Note that the square of *vF*^*T*^ defines the variance of execution noise, and like noise in sensor level, let *u*_*i*_ = *u*_*Ni*_ represent the final outputs of the proposed network.

## 4. Simulation Experiments

We conducted simulation experiments on the musculoskeletal system model to verify the performance of different algorithms. Moreover, the equilibria of action values are analyzed to explain the learning process of the proposed PTL framework.

### 4.1. Experimental Setup

As mentioned above, we designed a simplified musculoskeletal arm model to verify and evaluate the proposed learning method. After analyzing its dynamics (Equation 12), a basic control framework is devised. To validate the formulation and analyze performance, optimization should be performed.

In this study, the proposed PTL is applied to a point-to-point motion task with constant angular velocity as temporal-difference learning approach. For a final state of target motion, we calculated midpoints and required constraints using inverse kinematics. Then, we used joint angles as motion states to design the simplified target states. Assuming a constant angular velocity, four types of control strategies were evaluated: (1) Q-network, (2) Q-network with noises, (3) PTL, and (4) PTL with noises. The implemented method including PTL is detailed in Algorithm 1.

**Algorithm 1:** PTL with Noises for Motion Learning in Musculoskeletal System.

**Require:** Given precise motion states s(t)∈S . Initialize parameters: interval *d*, maximum number of iterations *K*, excitation signal *u*_*i*_. Obtain simplified motion state $s_{T}\left(t\right)\in S_{T}$ using Equation 13.
1: **for** k=1 **to** K **do**
2: Compute average allowed error ${\bar{e}}_{T}=\frac{1}{T}\overset{T}{\underset{t=1}{\sum }}|s\left(t\right)-s_{T}\left(t\right)|$
3: **if** ${\bar{e}}_{R}<{\bar{e}}_{T}$ and *k* ≠ 1 **then**
4: Reduce *d* gradually (*d* ∈ *N*_+_, *d*_max_ < *T*)
5: Set new target states $S_{T}$ by simplifying S
6: **end if**
7: **for** t=0 **to** T **do**
8: Calculate activation signal *a*_*i*_(*u*_*i*_(*t*)) and tendon force $F_{i}^{T}$
9: Perform motion corresponding to *s*_*R*_(*t* + 1) caused by $F_{i}^{T}$
10: Obtain actual motion error *e*_*R*_(*t*) = |*s*_*T*_(*t*) − *s*_*R*_(*t*)|
11: Introduce noise at sensor level into motion states via Equation 28. Let *s*_*R*_(*t*) = *s*_*RN*_(*t*) be the inputs of improved Q-network
12: Estimate $Q_{u_{i},j}^{\prime }$ by improved Q-network method
13: Update weights ω to obtain new action values *Q*_*u*_*i*_, *j*_ via Equation 23
14: Obtain signal *u*_*i*_(*t*) using Equation 18
15: Introduce noise at execution level into excitation signals via Equation 30. Let *u*_*i*_(*t*) = *u*_*Ni*_(*t*) be the outputs of improved Q-network
16: **end for**
17: **end for**

We set maximum number of iterations *K* = 500 and number of timesteps *T* = 10,000 to simulate 10 s. All the errors and control signals were recorded at each timestep.

### 4.2. Results and Analysis

We considered average error $\bar{e}=\frac{1}{T}\overset{T}{\underset{t=1}{\sum }}|\theta \left(t\right)-\theta_{R}\left(t\right)|$ as a key performance indicator, where θ(*t*) is the precise expected joint angle at time *t*. As $\bar{e}$ reflects the average error, motion performance can be evaluated from this measure.

**Figure 6:**
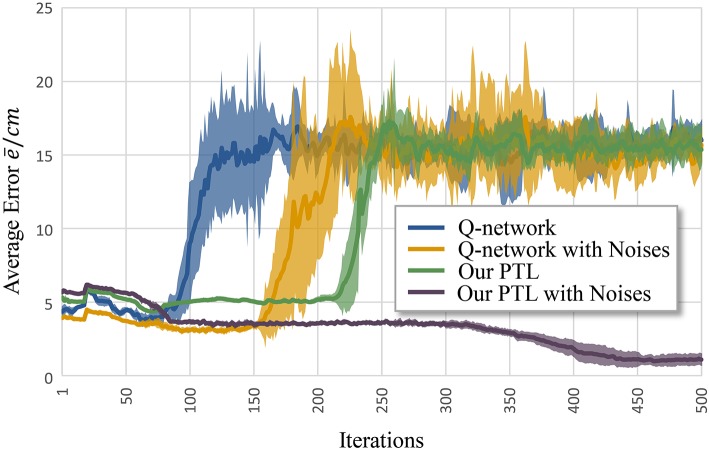
Average error for different methods to control musculoskeletal arm model for motion tracking. Curves correspond to average errors over 10 trials.

Figure 6 shows the average error $\bar{e}$ according to iteration *k*. Clearly, the Q-network method, Q-network with noises, and PTL are trapped at local optima and unstable during training. Still, phased targets improve learning by increasing the randomness of exploration, and noises during training enhance fault tolerance and the exploration ability during control.

Assume that the ratio of action-value functions is convergent to local optimum *b*_*i*_, which is defined as

(31) $$\begin{array}{r} b_{i}=\frac{Q_{u_{i},1}}{Q_{u_{i},2}}\;. \end{array}$$

Then, *u*_*i*_ can be rewritten as

(32) $$u_{i}=\frac{1}{\sum_{j=1}^{2}Q_{u_{i},j}}(Q_{u_{i},1}u_{\max}+Q_{u_{i},2}u_{\min})$$

(33) $$\begin{array}{r} =\frac{1}{b_{i}+1}\left(b_{i}u_{\max}+u_{\min}\right)\;, \end{array}$$

and hence the equilibrium point *b*_*i*_ is the only parameter that affects excitation signal *u*_*i*_. We prescribe that the control method adjusts *Q*_*u*_*i*_, 1_ and *Q*_*u*_*i*_,2_ in an opposite way. In addition, increment Δ*Q*_*u*_*i*_, *j*_ satisfies Δ*Q*_*u*_*i*_, *j*_ > 0 and Δ*Q*_*u*_*i*_, *j*_ ≪ *Q*_*u*_*i*_, *j*_ at simulation onset. The next equilibrium point at time (*t* + 1) is $b_{i}^{\prime }=\left(Q_{u_{i},1}\mp \Delta Q_{u_{i},1}\right)/\left(Q_{u_{i},2}\pm \Delta Q_{u_{i},2}\right)$, whose increment is given by

(34) $$\begin{array}{rc} b_{i}-b_{i}^{\prime } & =\frac{Q_{u_{i},1}}{Q_{u_{i},2}}-\frac{Q_{u_{i},1}\;\mp \;\Delta Q_{u_{i},1}}{Q_{u_{i},2}\;\pm \;\Delta Q_{u_{i},2}}\;, \end{array}$$

(35) $$\begin{array}{r} =\frac{\pm \left(Q_{u_{i},1}\Delta Q_{u_{i},2}\;+\;\Delta Q_{u_{i},1}Q_{u_{i},2}\right)}{Q_{u_{i},2}\left(Q_{u_{i},2}\;\pm \;\Delta Q_{u_{i},2}\right)}\;. \end{array}$$

For (−Δ*Q*_*u*_*i*_, 1_, +Δ*Q*_*u*_*i*_,2_), we obtain $b_{i}-b_{i}^{\prime }>0$, and excitation signal *u*_*i*_ becomes smaller. For (+Δ*Q*_*u*_*i*_, 1_, −Δ*Q*_*u*_*i*_,2_), as *Q*_*u*_*i*_,2_ − Δ*Q*_*u*_*i*_,2_ > 0, we obtain $b_{i}-b_{i}^{\prime }<0$, and excitation signal *u*_*i*_ becomes larger.

However, with reducing motion error, the increment of function *Q*_*u*_*i*_, *j*_ is smaller for *Q*_*u*_*i*_, *j*_ ≈ Δ*Q*_*u*_*i*_, *j*_. From Equation (35), when (+Δ*Q*_*u*_*i*_, 1_, −Δ*Q*_*u*_*i*_,2_), the sign of $\left(b_{i}-b_{i}^{\prime }\right)$ depends on the sign of (*Q*_*u*_*i*_,2_ − Δ*Q*_*u*_*i*_,2_). Nevertheless, it is difficult to guarantee either (*Q*_*u*_*i*_,2_ ⩽ Δ*Q*_*u*_*i*_,2_) or (*Q*_*u*_*i*_,2_ ⩾ Δ*Q*_*u*_*i*_,2_). The uncertain sign causes chattering on the excitation signal (Equation 33), which can cause signal divergence at the final state.

In addition, random factors like ϵ and noise can give rise to fluctuations of Δ*Q*_*u*_*i*_, *j*_, which may increase the adjustment extent. For example, if (+Δ*Q*_*u*_*i*_, 1_, +Δ*Q*_*u*_*i*_,2_) or (−Δ*Q*_*u*_*i*_, 1_, −Δ*Q*_*u*_*i*_,2_), the increment of *b*_*i*_ is given by

(36) $$\begin{array}{rc} b_{i}-b_{i}^{\prime } & =\frac{Q_{u_{i},1}}{Q_{u_{i},2}}-\frac{Q_{u_{i},1}\;\pm \;\Delta Q_{u_{i},1}}{Q_{u_{i},2}\;\pm \;\Delta Q_{u_{i},2}} \end{array}$$

(37) $$\begin{array}{r} =\frac{\pm \left(Q_{u_{i},1}\Delta Q_{u_{i},2}\;-\;\Delta Q_{u_{i},1}Q_{u_{i},2}\right)}{Q_{u_{i},2}\left(Q_{u_{i},2}\;\pm \;\Delta Q_{u_{i},2}\right)}\;, \end{array}$$

where (*Q*_*u*_*i*_, 1_Δ*Q*_*u*_*i*_,2_ − Δ*Q*_*u*_*i*_, 1_*Q*_*u*_*i*_,2_) with an uncertain sign can seriously undermine performance, as it is directly related to the sign of $\left(b_{i}-b_{i}^{\prime }\right)$. Furthermore, performance may decay even without condition *Q*_*u*_*i*_, *j*_ ≈ Δ*Q*_*u*_*i*_, *j*_, and the method will be unreliable under its influence. Fortunately, with appropriate training, performance degradation by random effects can almost be eliminated.

**Figure 7:**
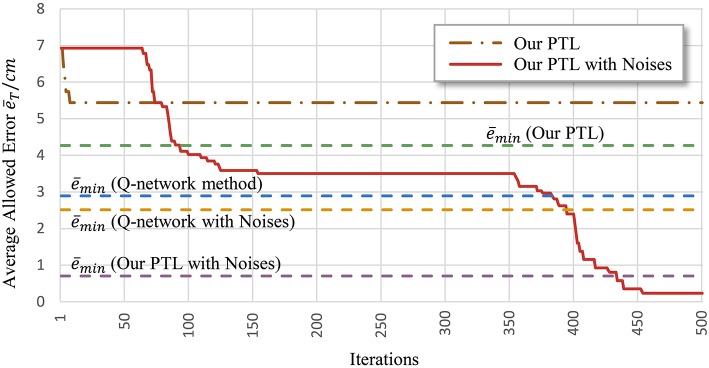
Average allowed error during training. Most algorithms stop learning before processing all the simplified targets.

Another problem is early convergence during learning. Figure 7 shows the evolution of the average allowed error. The four evaluated methods terminate searching when reaching different local optima. Generally, premature convergence occurs through the insufficient exploration of solutions. Given its exploration ability, the proposed PTL with noises was guided by simplified targets to avoid premature convergence. This method achieved the lowest error (average $\bar{e}<0.746cm$) and the most advanced learning level throughout repeated experiments.

(38) $$\begin{array}{r} b_{i}=\frac{Q_{u_{i},1}}{Q_{u_{i},2}}+\Delta b_{i}\; \end{array}$$

We define Δ*b*_*i*_ in Equation (38) as a small increment of the equilibrium point caused by the allowed error *e*_*T*_. As $\frac{Q_{u_{i},1}}{Q_{u_{i},2}}$ is not at the expected equilibrium point *b*_*i*_, *Q*_*u*_*i*_, *j*_ cannot easily generate large fluctuations. According to the analyses above, $\frac{Q_{u_{i},1}}{Q_{u_{i},2}}$ will converge to the final equilibrium point *b*_*i*_ when *t* = *T*.

**Figure 8:**
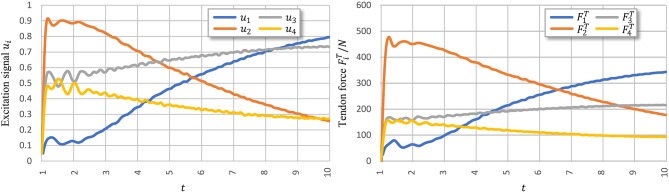
Execution signals trained using PTL with noises after 500 iterations. All excitation signals are filtered with a Butterworth lowpass filter to separate signals from execution noise.

**Figure 9:**
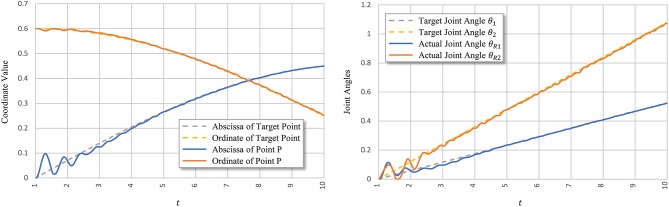
Tracking performance of PTL with noises. Point *P* is the terminal point for arm motion.

Figure 8 shows signal *u*_*i*_ learned using PTL with noises and the corresponding tendon force, $F_{i}^{T}$. Figure 9 shows the final position of the arm and joint angles. These results show that the most substantial errors occur at motion onset, and only slight fluctuations remain afterwards. At motion onset, it is reasonable to believe that unexpected muscle forces, especially passive forces of muscles 1 and 3, disturb the force balance. As the simulation proceeds, the arm model returns to a balance state by adjusting *u*_*i*_. Therefore, PTL extends learning and guides toward the next expected solutions. In addition, the noises foster an extensive exploration of the solution space during training.

**Figure 10:**
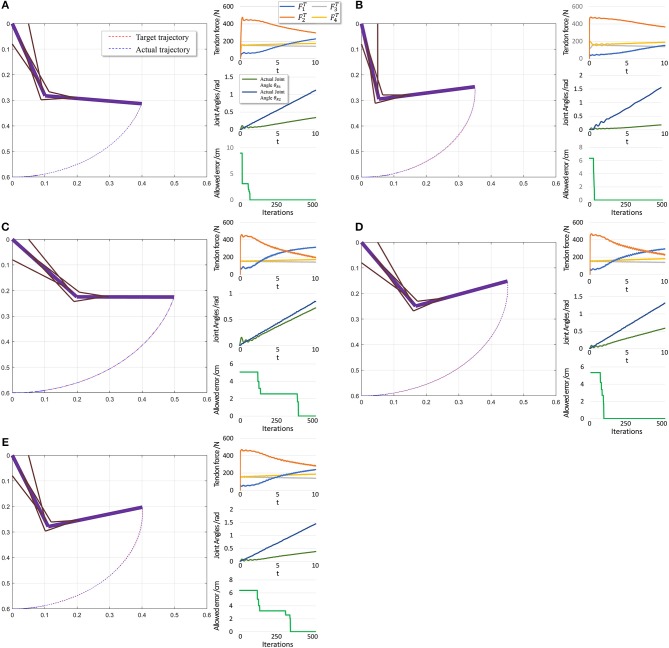
Scenario 1: Motion with constant angular velocity begins from a stable position and reaches another position in the motion space. **(A–E)** are five different trajectories selected randomly from operation space. Especially, all the initial states are the same (θ_1_ = 0, θ_2_ = 0). In each situation, Left: actual motion trajectory of endpoint achieved by PTL. Right: (Top) corresponding tendon forces caused by signal μ. (Middle) Actual joint angles during the motion. Remember that each trajectory task is required a constant angular velocity. (Bottom) Allowed error during training, which can be considered as phased target of motion learning.

To further evaluate PTL framework, we consider point-to-point motion through two scenarios. First, motion begins from a stable position (θ_*i*_ = 0) and finishes at another position (Figure 10).

When motion starts from a stable position, the next state *s*_*t*+1_ does not considerably change if *F*^*T*^ = 0. Therefore, the algorithm should not deal with large and rapid fluctuations, and the PTL performance is high. In contrast, in the second scenario, motion starts from an unstable position, and *s*_*t*+1_ exhibits a large difference when compared with *s*_*t*_ in the initial period even if *F*^*T*^ = 0, as the gravitational torque contributes to a large angular acceleration. Consequently, learning is unstable.

**Figure 11:**
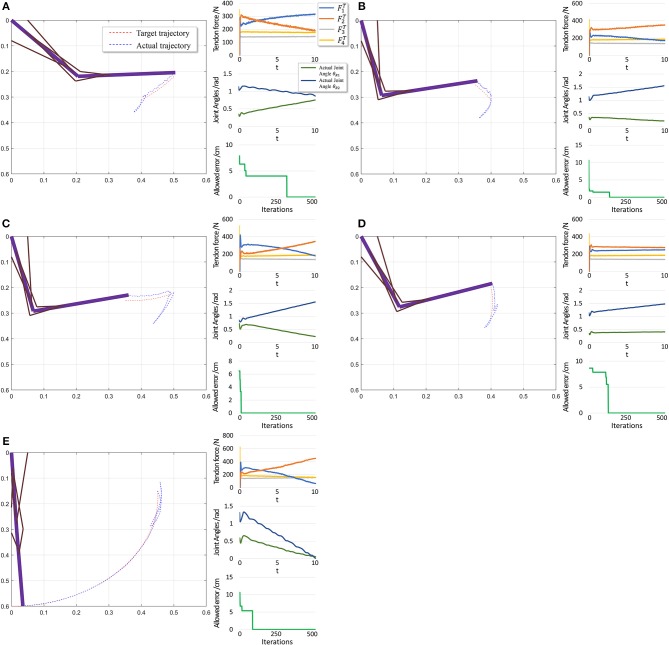
Scenario 2: Motion with constant angular velocity begins from an unstable position and reaches another position in the motion space. **(A–D)** are five different trajectories selected randomly from operation space. In each situation, Left: actual motion trajectory of endpoint achieved by PTL. Right: (Top) corresponding tendon forces caused by signal μ. (Middle) Actual joint angles during the motion. Remember that each trajectory task is required a constant angular velocity. (Bottom) Allowed error during training, which can be considered as phased target of motion learning. To evaluate performance, situation **(E)** is designed particularly to move from a unstable state to the stable position (*θ*_1_ = 0 *and θ*_2_ = 0).

The performance in the second scenario (Figure 11) confirms our prediction of large initial fluctuations. In fact, inappropriate initial parameters in musculotendon model will also degrade the performance. As inappropriate parameters lead to inappropriate muscle force, and some timesteps are necessary to adjust those parameters. In addition, the trajectory length is notably shorter than that in the first scenario, leading to a shorter trajectory for adjustment and learning. Consequently, errors increase in this scenario.

## 5. Conclusions

In this paper, we propose a human-inspired motion learning framework for a musculoskeletal system, called PTL. We analyze the learning process and equilibrium point of *Q*_*u*_*i*_, *j*_, determining that phased targets guide excitation signals toward expected values during learning. Two types of biological noise sources are considered in the PTL framework to increase the exploration ability in an expanded solution space, making the algorithm suitably follow the guidance of phased targets. Theoretically, as PTL is based on a human learning process, it can be expanded as a general-purpose learning framework if we find appropriate ways to simplify different kinds of tasks, such as capture and pattern recognition tasks.

In future work, we will apply advanced methods in PTL to improve performance, especially when motion starts from an unstable position. Furthermore, better approaches for simplifying tasks and more biological mechanisms of motion control should be investigated to expand the application scope of the PTL framework.

## Data Availability

All datasets generated for this study are included in the manuscript and/or the supplementary files.

## Author Contributions

JZ provided the main ideas of this research, wrote the manuscript and codes of experiments. JC and HD provided suggestions about PTL framework. HQ and other authors discussed and revised the manuscript.

### Conflict of Interest Statement

The authors declare that the research was conducted in the absence of any commercial or financial relationships that could be construed as a potential conflict of interest.
